# A novel, major, and validated QTL for grain zinc concentration independent of yield traits in tetraploid wheat

**DOI:** 10.1002/tpg2.70029

**Published:** 2025-04-23

**Authors:** Zhaoyong Zeng, Jian Ma, Bingjie Chen, Huaping Tang, Xin Xian, Yuanfeng Huo, Yinggang Xu, Xiaoyan Tang, Xuesong Gao, Guangdeng Chen

**Affiliations:** ^1^ College of Resources Sichuan Agricultural University Chengdu China; ^2^ Triticeae Research Institute Sichuan Agricultural University Chengdu China

## Abstract

Zinc deficiency is a critical global health issue, with declining grain zinc concentration (GrZnc) over time. To address this, it is essential to explore and utilize genetic resources from wild relatives to enhance GrZnc in cultivated bread wheat (*Triticum aestivum* L.). This study aimed to identify quantitative trait loci (QTL) for GrZnc using a recombinant inbred line population (AM population), derived from a cross between the Sichuan‐endemic tetraploid wheat Ailanmai and wild emmer accession LM001. A linkage map was constructed based on the wheat 55K single‐nucleotide polymorphism array, and phenotypic data were collected from five different environments. Four QTL for GrZnc were identified, spanning three chromosomal regions. Notably, a novel and stable QTL, *QGrZnc.sau‐AM‐4A*, was detected in all environments and the best linear unbiased prediction dataset. This QTL, with LOD values ranging from 2.72 to 9.31, explained 12.31%–30.50% of the phenotypic variance and was mapped to chromosome arm 4AS (54.43–60.02 Mbp). Interestingly, this QTL had no significant effect on key agronomic traits such as spike length, 1000‐kernel weight, kernel number per spikelet, spikelet number per spike, spike density, and plant height, indicating no dilution effects. A kompetitive allele‐specific PCR (KASP) marker, *KASP‐AX‐108829087*, closely linked to this major QTL, was developed and validated in two different genetic populations. A candidate gene (*TRIDC4AG008520*) related to zinc absorption and transport was identified within the *QGrZnc.sau‐AM‐4A* interval. These findings provide insight into the genetic basis of GrZnc and establish a foundation for further fine mapping and map‐based cloning of this locus.

## Core Ideas

- This study identifies quantitative trait loci (QTL) for grain zinc concentration in tetraploid wheat.
- QTL were identified using a recombinant inbred line population and a wheat 55K single‐nucleotide polymorphism array‐based constructed genetic map.
- The genetic effects of the major and stable QTL for grain zinc concentration was validated in genetic backgrounds.
- Tightly linked kompetitive allele‐specific PCR (KASP) marker (*KASP‐AX‐108829087*) can be further used in molecular marker‐assisted selection.

**Abbreviations**
BLUP: best linear unbiased prediction
CWL: Chinese wheat landraces
GrZnc: grain zinc concentration
GY: grain yield
KASP: kompetitive allele‐specific PCR
KNS: kernel number per spikelet
MAS: marker‐assisted selection
QTL: quantitative trait loci
RIL: recombinant inbred line
SD: spike density
SL: spike length
SNP: single‐nucleotide polymorphism
SNS: spikelet number per spike
TKW: 1000‐kernel weight

## 1 BACKGROUND

Wheat (*Triticum aestivum* L.) is one of the most widely cultivated crops, supplying nearly a quarter of the global caloric and protein intake. Since the Green Revolution, wheat production has increased significantly (Evenson & Gollin, 2003). However, modern wheat varieties, which have replaced traditional cultivars, often contain lower micronutrient levels (Welch, 2002). As a result, grain zinc concentration (GrZnc) has become a major global health concern, affecting over 700 million children and women (Sun et al., 2023). Several strategies for improving human nutrition have been proposed to address this, including dietary diversification, supplementation, fortification, and biofortification (Ali & Borrill, 2020). Among these, biofortification is a cost‐effective and efficient solution to combat malnutrition.

To meet the human body's zinc requirements, the zinc concentration in whole grains should be around 37 mg/kg (Meena et al., 2020). However, current wheat cultivars in China have a GrZnc of only 30 mg/kg (Liu et al., 2014). Wild emmer wheat, the ancestor of modern tetraploid and hexaploid wheat, exhibits high zinc levels within the *Triticum* species (Peng et al., 2003; Yu et al., 2020). Wild emmer plays a crucial role in enhancing GrZnc in bread wheat breeding (Cakmak et al., 2004; Gomez‐Becerra et al., 2010). Additionally, landrace wheat varieties, preserved through long‐term natural and human selection, represent unique genetic resources (Crossa et al., 2016). Therefore, leveraging the genetic potential of wild emmer and wheat landraces is essential for addressing zinc deficiency in wheat breeding.

Several quantitative trait loci (QTL) associated with GrZnc and related traits in wheat have been reported. For example, six QTL for zinc concentration at the seedling stage were mapped on chromosomes 2D, 4A, 4D, 5B, 5D, and 6A (Genc et al., 2009). Other studies identified two QTL on chromosomes 4B and 5A (Xu et al., 2012), two major QTL on chromosomes 1B and 6B (Velu et al., 2017), and seven QTL on chromosomes 1D, 2A, 3B, 4D, 6A, 6D, and 7B (Wang et al., 2021). More recently, 16 QTL have been detected on chromosomes 1B, 2B, 2D, 3A, 3D, 4A, 4B, 5A, 5D, 6B, and 7D (Liu et al., 2021), and eight QTL on chromosomes 1B, 2D, 4B, 4D, 5A, 5D, 6A, 6D, and 7B (Sun et al., 2023). However, only a few major QTL for GrZnc have been stably validated across multiple environments with different genetic backgrounds, and high‐efficiency molecular markers are still limited. To date, the only successfully cloned gene influencing GrZnc is *Gpc‐B1*, which has been incorporated into breeding programs (Distelfeld et al., 2007; Uauy et al., 2006). This highlights the need for further research to uncover the genetic basis and regulatory mechanisms of GrZnc in wheat.

In this study, a tetraploid recombinant inbred line (RIL) population derived from a cross between the Sichuan‐endemic tetraploid wheat Ailanmai and the wild emmer accession LM001 was used to identify QTL for GrZnc. The linkage maps were constructed using a wheat 55K single‐nucleotide polymorphism (SNP) array, and phenotypic data from five different environments were analyzed. The objectives were (a) to identify genetic loci associated with GrZnc and (b) to validate the major QTL across different genetic backgrounds using a new kompetitive allele‐specific PCR (KASP) marker derived from the identified QTL. These findings provide a solid foundation for selecting wheat lines with desirable GrZnc traits using molecular markers and for further fine mapping and map‐based cloning of the novel QTL.

## 2 MATERIALS AND METHODS

### 2.1 Genetic populations

A RIL population of tetraploid wheat, consisting of 119 F_9_ lines, was developed through hybridization between the Sichuan landrace Ailanmai (AL, *Triticum turgidum* L., 2*n* = 28, AABB) and wild emmer (LM001, *T*. *turgidum* subsp. *dicoccoides*, 2*n* = 28, AABB) (Mo et al., 2021). AL is a durum wheat landrace known for its multiple florets and strong adaptability to varying environmental conditions (Liu et al., 1999). LM001 is characterized by long awns and non‐free threshability. Additionally, a separate RIL population, consisting of LM001 × PI503554 (*T. turgidum* ssp. *dicoccon*) with 102 F_3_ lines, and a panel of Chinese wheat landraces (CWL), were used to study the effects of positive alleles from the major QTL. All plant materials were provided by the Triticeae Research Institute of Sichuan Agricultural University.

### 2.2 Phenotypic evaluation

The RILs, along with their parental lines, were planted across five different field locations: Wenjiang (103°51′ E, 30°43′ N) from 2020 to 2022 (2020WJ, 2021WJ, and 2022WJ), Ya'an (103°0′ E, 29°58′ N) in 2020 (2020YA), and Chongzhou (103°38′ E, 30°32′ N) in 2022 (2022CZ). The LM001 × PI503554 population and CWL were planted in Chongzhou in 2021. Soil characteristics prior to planting are detailed in Table S1.

A randomized complete block design with two replications was employed at each site. Each line, consisting of 15 plants, was sown in a single 1.5‐m row, with 0.1‐m spacing between plants and 0.3‐m spacing between rows. Fertilization was applied at rates of 80 kg/ha for nitrogen and 100 kg/ha for potassium phosphate. Field management followed standard wheat cultivation practices. At physiological maturity, grains from two plants per line were harvested separately as replicates, with three replications per line. Grains were hand‐threshed, cleaned, weighed, crushed, and sieved. A 0.1‐ to 0.2‐gram aliquot of the sieved sample was mixed with 5 mL of a 4:1 HNO₃:HClO₄ mixture and heat‐digested at 220°C for 4–5 h until the solution became colorless and transparent. The digested solution was reduced to 1–2 mL, cooled, and then adjusted to 5 mL with 1% HNO₃ and filtered. GrZnc was measured using ICP‐MS (PerkinElmer Nexlon2000).

**Core Ideas**

- This study identifies quantitative trait loci (QTL) for grain zinc concentration in tetraploid wheat.
- QTL were identified using a recombinant inbred line population and a wheat 55K single‐nucleotide polymorphism array‐based constructed genetic map.
- The genetic effects of the major and stable QTL for grain zinc concentration was validated in genetic backgrounds.
- Tightly linked kompetitive allele‐specific PCR (KASP) marker (*KASP‐AX‐108829087*) can be further used in molecular marker‐assisted selection.

The best linear unbiased prediction (BLUP) datasets for plant height (PH), spike length (SL), 1000‐kernel weight (TKW), kernel number per spikelet (KNS), spikelet number per spike (SNS) (Mo et al., 2021), spike density (SD) (You et al., 2021), and grain yield (GY) from the AM population were analyzed for Pearson's correlation and subjected to Student's *t*‐test. Detailed environmental data for these traits are provided in Table S2.

### 2.3 Data analysis

SAS 8.0 (SAS Institute; https://www.sas.com) was used to calculate BLUP and broad‐sense heritability (*H* ^2^) of GrZnc across various environments (Smith et al., 1998). The formula for determining *H* ^2^ is as follows: *H* ^2^ **= *V*_G_/(*V*_G_ + *V*_E_), where *V*_G_ is genetic variance and *V*_E_ denotes environmental variance. The BLUP was computed utilizing the model: Yi = Xif + ai + ei, where *f* is a fixed‐effects vector, Xi is an incidence vector, ai is the value of phenotype, and ei indicates the environmental deviation. IBM SPSS Statistics v27 (SPSS; http://en.wikipedia.org/wiki/SPSS) was used to perform analysis of variance (ANOVA), independent‐sample *t*‐test (*p *< 0.05), and Pearson correlation coefficients. OriginPro 2024 (https://www.originlab.com/getstarted) was used to draw boxplots, correlation plots, and frequency distributions. IciMapping 4.1 (https://www.isbreeding.net/) was used for ANOVA of multi‐environmental trials.

### 2.4 QTL mapping

QTL analysis was performed using the genetic linkage map constructed with the 55K SNP array, spanning 2,411.84 cM with an average marker distance of 2.10 cM and comprising 1,150 bin markers (Mo et al., 2021). QTL were identified using the inclusive composite interval mapping method within IciMapping 4.1. The threshold parameters were set at 1.0 cM, PIN value 0.001, and LOD value 2.5. QTL identified in more than three environments and explaining over 10% of the phenotypic variance were considered stable (Zeng et al., 2023; Zeng et al., 2024b).

Conditional QTL analysis, which reveals complex trait relationships, was also performed. A significant increase or decrease in LOD values indicates partial or full dependency on other traits (Cui et al., 2011). Conditional phenotypic values for GrZnc|PH and GrZnc|TKW were calculated using QGAStation2.0 software (http://ibi.zju.edu.cn/software/), and conditional QTL mapping was done with IciMapping V4.1. QTL were named following the International Rules of Genetic Nomenclature, where “sau” refers to Sichuan Agricultural University and “AM” denotes the mapping population.

### 2.5 Marker development and QTL validation

A tightly linked marker, *AX‐108829087*, associated with the major QTL was converted into a KASP marker (*KASP‐AX‐108829087*) to validate the genetic effects of the major QTL (Table S3). The KASP reaction mixture consisted of 0.75 µL template DNA, 1.4 µL primer mix, 2.85 µL deionized water, and 5 µL SsoFast EvaGreen mixture (You et al., 2021). PCR conditions included 15 min at 94°C, followed by 40 cycles of 20 s at 94°C and 60 s at 61°C–55°C (dropping 0.6°C per cycle). Real‐time PCR was performed using a Bio‐Rad CFX‐96 system (Mo et al., 2021; Zeng et al., 2024a).

A set of 100 lines from the CWL and LM001 × PI503554 populations were genotyped and divided into two groups based on homozygous alleles from AL or LM001. GrZnc differences between the groups were assessed using Student's *t*‐test (*p *< 0.05).

### 2.6 Physical intervals of major QTL and comparison with previous studies

The sequences of the flanking markers closely linked to confidence interval of QTL for GrZnc on chromosome 4A were retrieved by BLASTing against (*E*‐value of 1 × 10^−5^) genomes of wild emmer (accession Zavitanv2.0) (Zhu et al., 2019) and Chinese Spring (CS) (IWGSC RefSeq v2.1) (Zhu et al., 2021). The same method was used to determine the physical locations of previously reported genes or QTL related to Zn traits on 4AS. Candidate gene annotations and functions were obtained from UniProt (http://www.uniprot.org/) and WheatOmics (http://202.194.139.32/). Gene expression data were retrieved from the tetraploid wheat expression database (http://202.194.139.32/ expression/emmer.html) and hexaploid wheat expression database (http://202.194.139.32/expression/wheat.html) of WheatOmics.

## 3 RESULTS

### 3.1 Phenotypic data analyses

**FIGURE 1:**
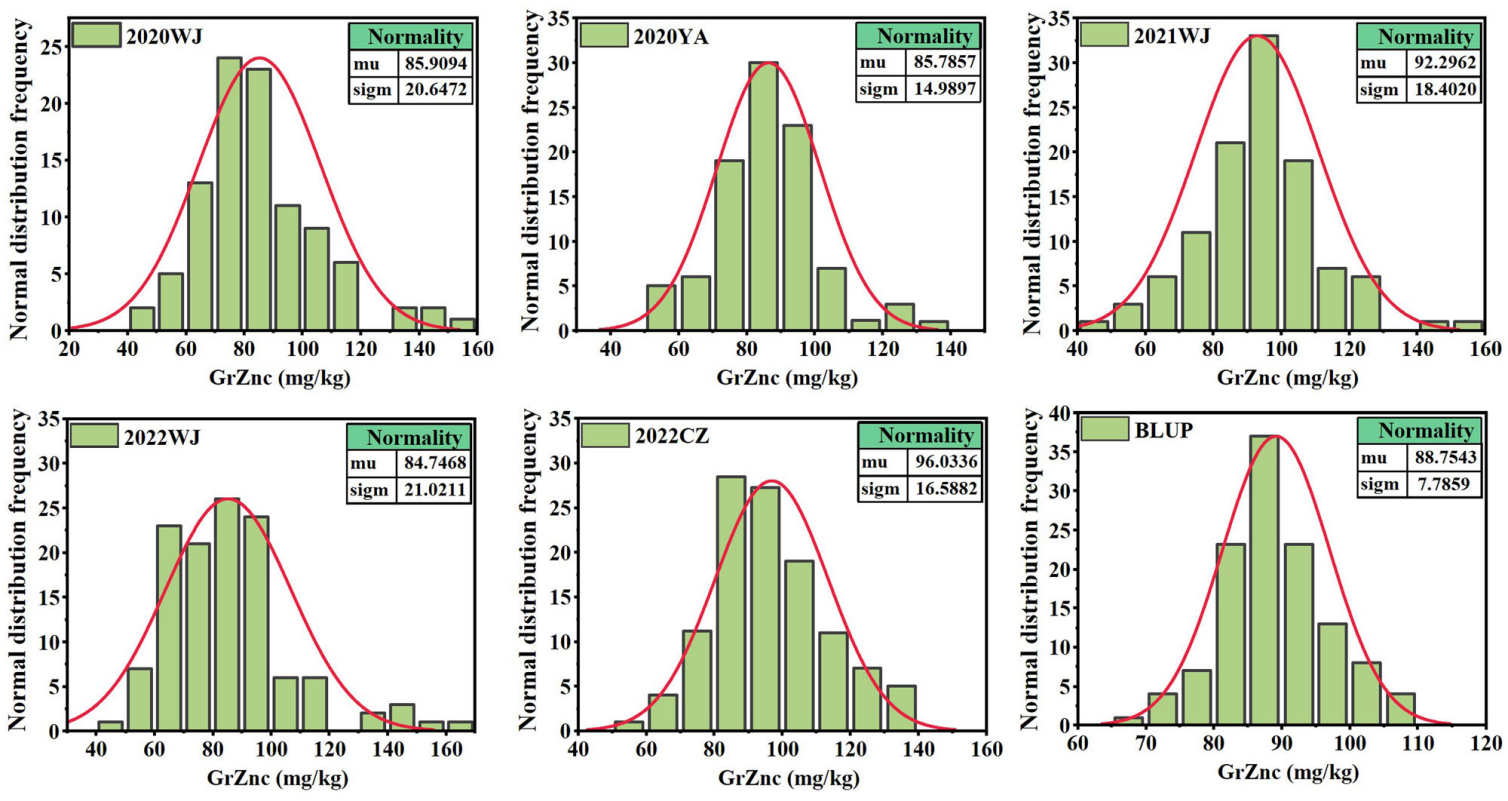
Frequency distributions of grain zinc concentration (GrZnc) from different environments. BLUP, best linear unbiased prediction; CZ, Chongzhou; WJ, Wenjiang; YA, Ya'an.

**FIGURE 2:**
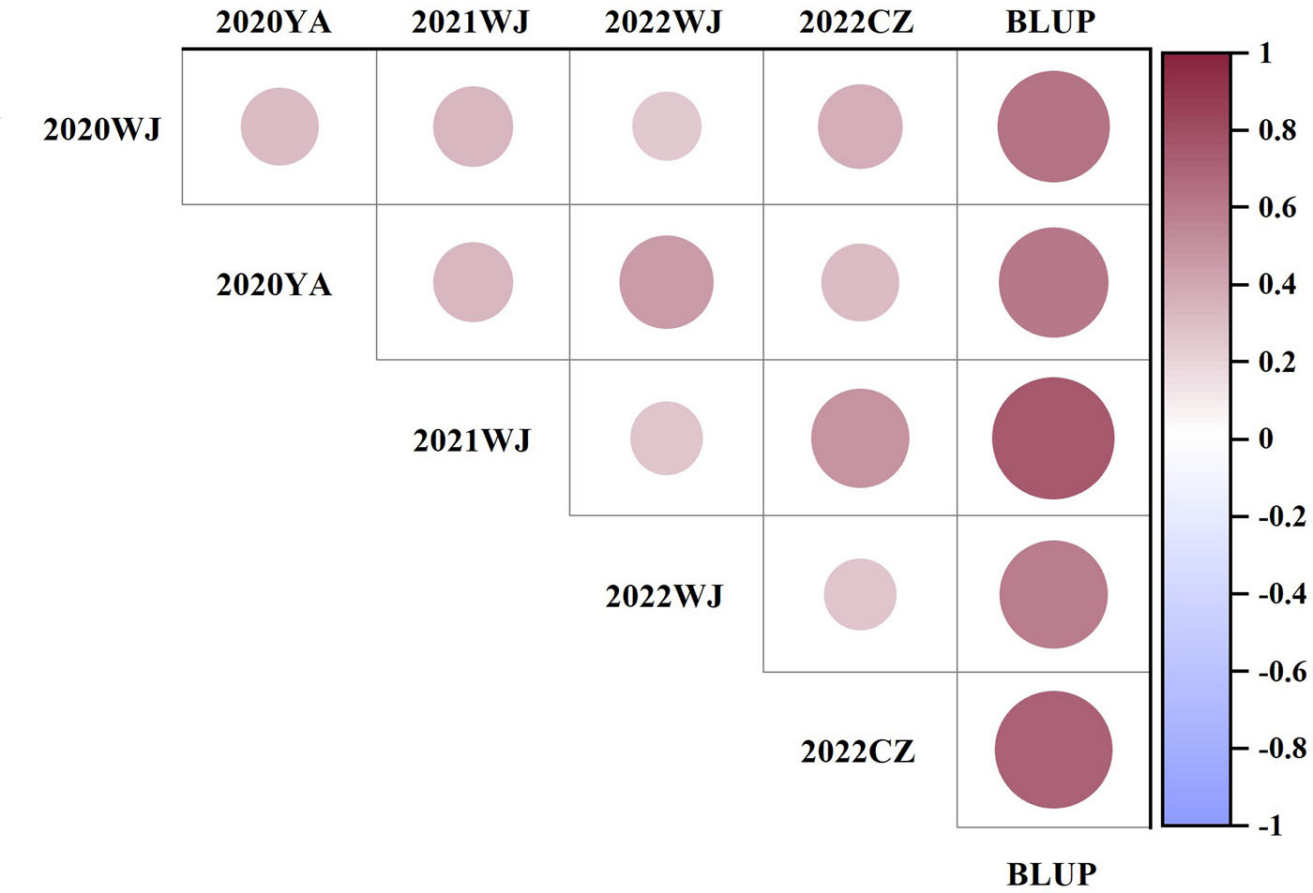
The correlation coefficients of grain zinc concentration (GrZnc) in multiple environments. BLUP, best linear unbiased prediction; CZ, Chongzhou; WJ, Wenjiang; YA, Ya'an.

LM001 exhibited significantly higher GrZnc than AL across all environments (*p* < 0.01). The GrZnc values for AL ranged from 61.76 to 105.19 mg/kg, while those for LM001 ranged from 80.76 to 115.19 mg/kg (Table 1). The AM population showed a GrZnc range from 43.51 to 162.02 mg/kg, with mean values of 82.82, 83.40, 92.30, 84.75, and 96.03 mg/kg across five environments, respectively (Table 1). The coefficient of variation ranged from 8.55% to 31.21%, with a heritability (*H* ^2^) of 0.53. GrZnc displayed a continuous distribution, closely approximating a normal distribution in all environments, suggesting a polygenic inheritance pattern (Figure 1). Furthermore, significant positive correlations for GrZnc were observed across all five environments (Figure 2).

**TABLE 1:** Phenotypic variation of grain zinc concentration (GrZnc) from five environments in the AM population.

Trait	Environment	Parents		RILs			
		AL	LM001	Min–max	Mean	CV (%)	*H* ^2^
GrZnc (mg/kg)	2020WJ	61.76	80.76**	45.56–157.15	82.82 ± 23.64	28.54	0.53
	2020YA	88.70	97.97**	52.50–131.63	83.4 ± 26.03	31.21	
	2021WJ	105.19	115.19**	43.51–159.78	92.3 ± 17.29	18.73	
	2022WJ	85.66	104.84**	46.87–162.02	84.75 ± 19.87	23.75	
	2022CZ	100.86	113.66**	56.34–138.22	96.03 ± 16.18	16.85	
	BLUP	88.54	97.82	67.61–109.36	88.75 ± 7.59	8.55	

Abbreviations: Al, Ailanmai; BLUP, best linear unbiased prediction; CV, coefficient of variation; CZ, Chongzhou; *H* ^2^, broad‐sense heritability; RIL, recombinant inbred line; SD, standard deviation; WJ, Wenjiang; YA, Ya'an.

* and ** mean significant difference at *p* < 0.05 and 0.01 probability level.

### 3.2 Correlation analysis between GrZnc and other agronomic traits

We examined the phenotypic correlations between GrZnc and several agronomic traits (SL, SD, SNS, KNS, TKW, and GY) based on BLUP data. The Pearson's correlation coefficients ranged from −0.15 to 0.08 (Figure S1). No significant correlations were found between GrZnc and SL, SD, SNS, KNS, TKW, or GY. ANOVA revealed that environment (E), genotype (G), and their interaction (G × E) significantly influenced GrZnc (Table S4).

### 3.3 Identification of QTL for GrZnc

**FIGURE 3:**
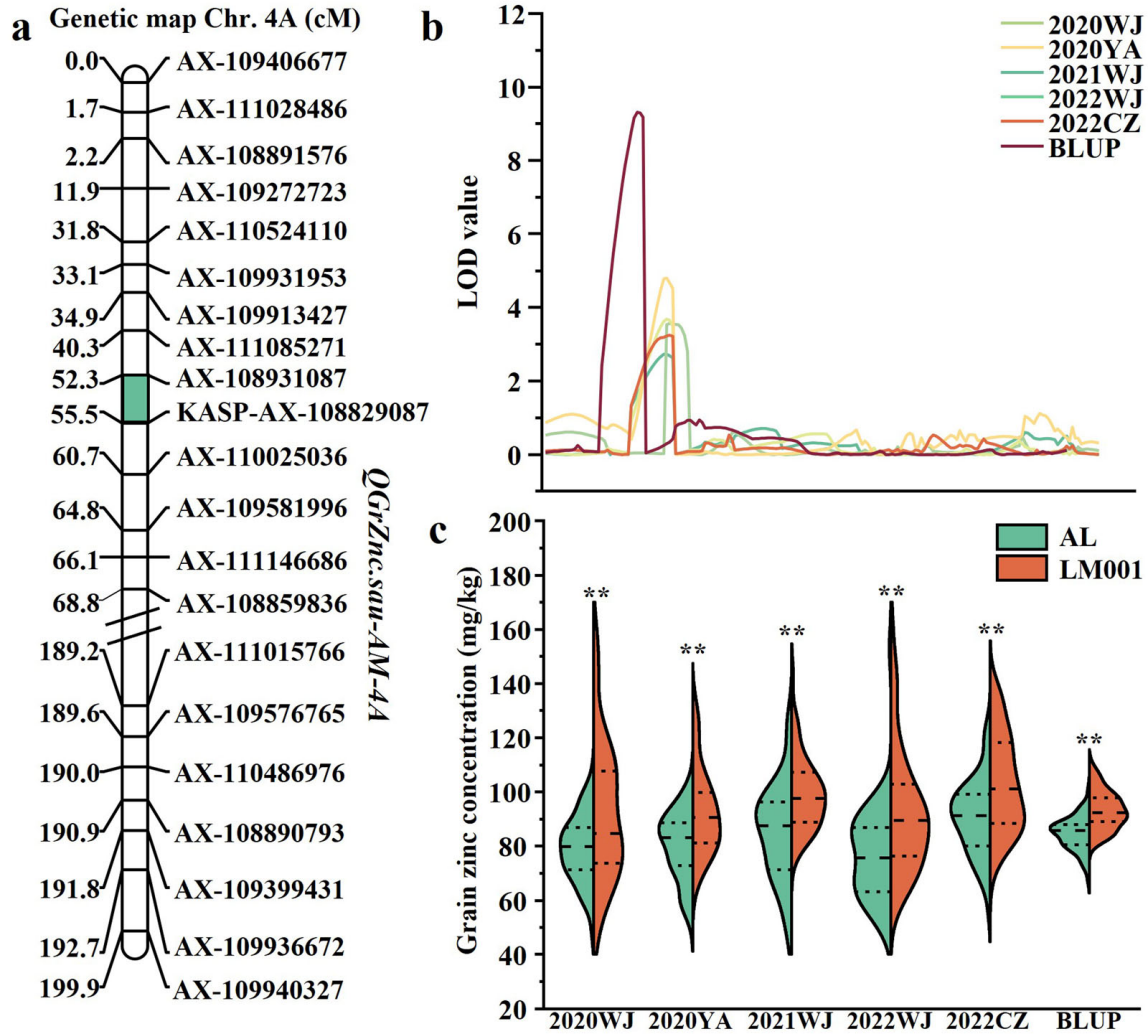
Genetic map of the major quantitative trait loci (QTL). (a) Genetic map of the QTL for grain zinc concentration (GrZnc) identified in AM population. (b) Log of odds (LOD) value of QTL for GrZnc of chromosome 4A. (c) AM population were divided into two haplotype groups based on the genotype of flanking markers, and the GrZnc differences caused by the corresponding QTL were represented. **Significance level at *p* < 0.01. BLUP, best linear unbiased prediction; CZ, Chongzhou; WJ, Wenjiang; YA, Ya'an.

Four QTLs for GrZnc were identified, located on chromosomes 4A (1 QTL), 7A (1 QTL), and 7B (2 QTLs), with LOD scores ranging from 2.72 to 9.31 (Table 2). These QTLs accounted for 10.65%–30.50% of the phenotypic variation. *QGrZnc.sau‐AM‐4A* emerged as a major locus, identified consistently across all environments and the BLUP dataset. It was mapped between *AX‐108931087* and *AX‐108829087* on chromosome 4A, explaining 12.72%–32.14% of the phenotypic variation, with LOD values ranging from 2.72 to 9.31 (Figure 3; Table 2). The positive allele of *QGrZnc.sau‐AM‐4A* was inherited from LM001. *QGrZnc.sau‐AM‐7A* and *QGrZnc.sau‐AM‐7B.2* exhibited LOD values of 3.62 and 2.77, accounting for 10.33% and 10.65% of the phenotypic variance, respectively, but were detected in only one environment (Table 2). Their positive alleles were derived from the parent LM001. *QGrZnc.sau‐AM‐7B.1* was detected in the 2020YA environment and accounted for 11.65% of the phenotypic variance, with its allele derived from AL. These QTLs were classified as minor.

**TABLE 2:** Quantitative trait loci (QTL) for grain zinc concentration (GrZnc) detected in the AM population.

Trait	QTL	Environments	Chromosome	Position	Left marker	Right marker	LOD	PVE (%)	Add
GrZnc	*QGrZnc.sau‐AM‐4A*	2020WJ	4A	53	*AX‐108931087*	*AX‐108829087*	2.72	12.31	−7.16
		2020YA	4A	54	*AX‐108931087*	*AX‐108829087*	3.56	15.39	−5.62
		2021WJ	4A	53	*AX‐108931087*	*AX‐108829087*	3.68	14.67	−6.58
		2022WJ	4A	53	*AX‐108931087*	*AX‐108829087*	4.80	14.06	−8.20
		2022CZ	4A	54	*AX‐108931087*	*AX‐108829087*	3.25	12.43	−5.84
		BLUP	4A	53	*AX‐108931087*	*AX‐108829087*	9.31	30.50	−4.26
	*QGrZnc.sau‐AM‐7A*	2022WJ	7A	133	*AX‐108805298*	*AX‐109902226*	3.62	10.33	−7.03
	*QGrZnc.sau‐AM‐7B.1*	2020YA	7B	68	*AX‐94418646*	*AX‐109897967*	2.76	11.65	4.87
	*QGrZnc.sau‐AM‐7B.2*	2021WJ	7B	191	*AX‐109988988*	*AX‐109403944*	2.77	10.65	−5.63

Abbreviations: Add: additive effect of a QTL; BLUP, best linear unbiased prediction; CZ, Chongzhou; LOD, logarithm of odds; PVE, phenotype variance explained; WJ, Wenjiang; YA, Ya'an.

**FIGURE 4:**
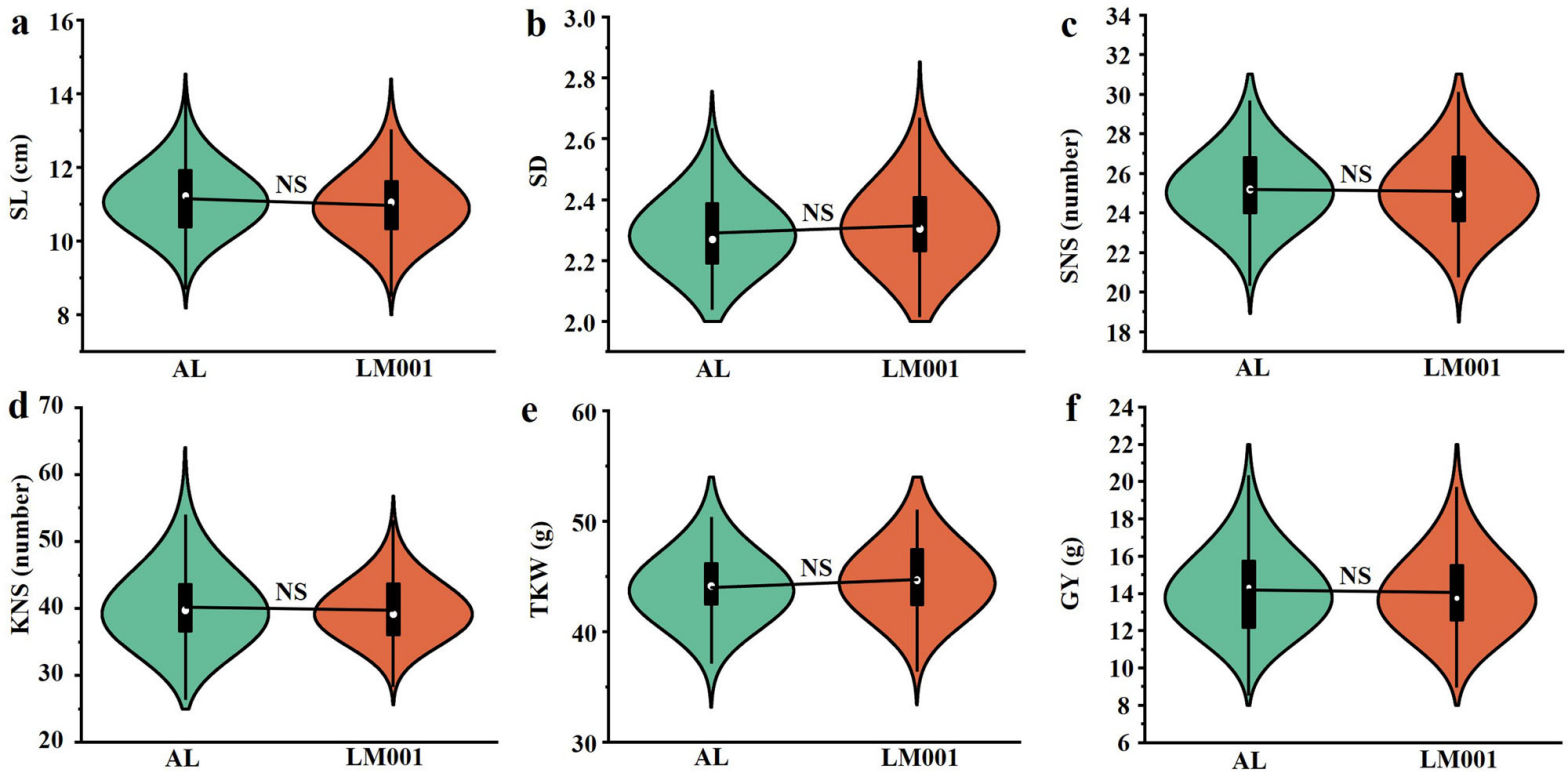
Student's *t*‐test for the two groups of lines carrying the allele from either AL or LM001 at *QGrZnc.sau‐AM‐4A* from the AM recombinant inbred line (RIL) population for spike length (SL), (a), spike density (SD), (b), spikelet number per spike (SNS), (c), kernel number per spikelet (KNS), (d), 1000‐kernel weight (TKW), (e), and grain yield (GY), (h). + and − represent lines with and without the positive alleles of *QGrZnc.sau‐AM‐4A* based on the flanking markers *KASP‐AX‐108829087* (where *KASP* is like kompetitive allele‐specific PCR).

Based on the flanking markers of *QGrZnc.sau‐AM‐4A*, the AM population was divided into two groups: one with homozygous alleles from AL, and the other with homozygous alleles from LM001. No significant differences were observed among these groups in SL, SD, SNS, KNS, TKW, or GY (Figure 4).

### 3.4 Validation of the major QTL *QGrZnc.sau‐AM‐4A*

**FIGURE 5:**
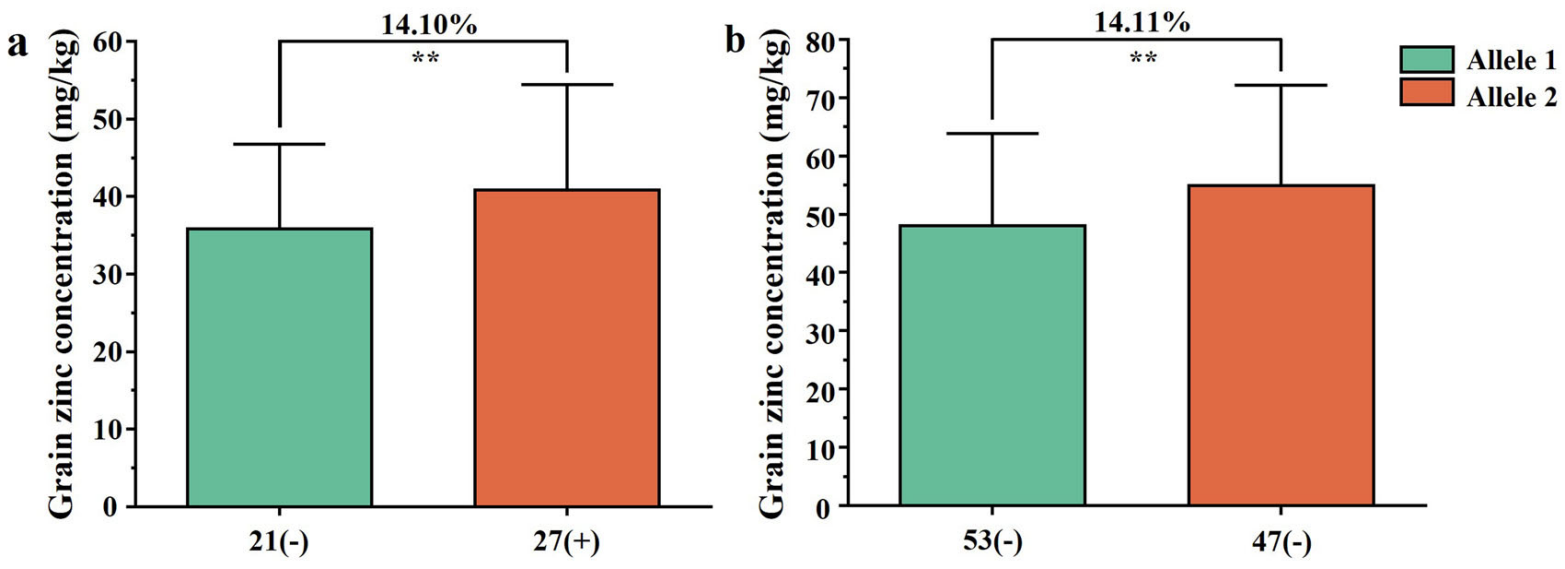
Validation of *QGrZnc.sau‐AM‐4A* in populations of LM001/PI503554 (MP) and Chinese wheat landraces. Allele 1 (−) and Allele 2 (+) represent lines without and with the positive alleles of the target quantitative trait loci (QTL) based on the flanking marker. **Significance level at *p* < 0.01, respectively.

The newly designed KASP marker *KASP‐AX‐108829087*, closely linked to *QGrZnc.sau‐AM‐4A*, was used to assess the effect of this locus in the LM001/PI503554 population and CWL. In both populations, 48 and 100 homozygous lines were grouped based on their genotyping results, distinguishing between lines with homozygous alleles from AL and LM001. Lines possessing the positive allele of *QGrZnc.sau‐AM‐4A* from LM001 exhibited significantly higher GrZnc levels than those with the negative allele from AL. These lines showed a 14.10% and 14.11% increase in GrZnc in the LM001/PI503554 population and CWL, respectively (Figure 5).

## 4 DISCUSSION

### 4.1 Multiple environment‐based analyses of GrZnc

GrZnc is a complex quantitative trait, highly sensitive to environmental factors. The concentration of extractable metal forms in the soil is influenced by variables such as pH, organic matter, and cation exchange capacity (Baquy et al., 2018; Martínez & McBride, 2001; Zhang, et al., 2021). These factors can lead to uneven distribution of Zn across agricultural fields, thus impacting GrZnc in wheat. Therefore, obtaining phenotypic data across multiple environments is essential to enhance the reliability of QTL findings. In this study, we identified a stable and major QTL, *QGrZnc.sau‐AM‐4A*, on chromosome arm 4AS, which was consistent across different soil types, years, and environments. Moreover, a novel KASP marker was developed for this QTL, which will be crucial in accelerating the development of biofortified crops.

### 4.2 *QGrZnc.sau‐AM‐4A* as a novel and major QTL

To explore the relationship between *QGrZnc.sau‐AM‐4A* and previously identified QTL on chromosome 4A, we compared their physical positions on the CS reference genome. There are limited reports of QTLs for Zn‐related traits on chromosome 4A in wheat. For example, *QGZn.cimmyt‐4A‐P2* (123.5–124.5 Mbp) for GrZnc was mapped to chromosome arm 4AS (Crespo‐Herrera et al., 2017). In contrast, *QGrZnc.sau‐AM‐4A* was mapped between 60.02 and 78.86 Mbp on chromosome arm 4AS, distinct from previously reported Zn‐related loci, suggesting that it may represent a novel QTL for GrZnc in wheat.

The stable and major *QGrZnc.sau‐AM‐4A* was mapped between markers *AX‐108931087* and *AX‐108829087*. The physical locations of these markers in the CS 2.1 and wild emmer V2 reference genomes were 60.96–78.86 Mbp and 54.43–60.02 Mbp, respectively. According to the wild emmer reference genome, there are 44 candidate genes within the interval of *QGrZnc.sau‐AM‐4A* (Figure S2). Analysis of the spatiotemporal expression patterns and functional annotations of these genes suggested that *TRIDC4AG008520* might be involved in the absorption and transport of Zn (Figure S3). Previous studies have shown that the severity of Zn deficiency symptoms correlates with the expression of copper/zinc superoxide dismutase (Cu/Zn SOD) (Fariduddin et al., 2022; Hacisalihoglu et al., 2004). Elevated Cu/Zn SOD levels help protect plant tissue from oxidative stress (Cakmak, 2000).

### 4.3 *QGrZnc.sau‐AM‐4A* is independent of the dilution effect

Since the “Green Revolution,” the introduction of genes controlling PH has contributed to a decrease in GrZnc levels (Velu & Singh, 2019; Wang et al., 2020). For instance, the *Rht‐B1b* allele reduces PH and enhances dry matter accumulation in favorable environments. However, the transfer of minerals to grains does not align with the redistribution of photosynthates, resulting in a “dilution effect” (Fan et al., 2008). RILs with the semi‐dwarf allele (*Rht‐B1b*) tend to exhibit higher productivity but lower GrZnc compared to those without it (Luo et al., 2021). Some pleiotropic QTLs for GrZnc are positively correlated with PH (Crespo‐Herrera et al., 2016; Wang et al., 2021), suggesting that mineral content in grains is influenced by PH.

In this study, conditional QTL analysis revealed that the major QTL *QGrZnc.sau‐M‐4A* on chromosome 4AS is independent of both PH and TKW (Figures S4 and S5). No genes or QTLs for PH were reported in this interval (Agarwal et al., 2020; Mo et al., 2021; Xu et al., 2023). Interestingly, *QGrZnc.sau‐AM‐4A* showed no significant correlation with any agronomic traits evaluated (Figure 4), indicating that it likely does not induce a dilution effect.

### 4.4 Application in wheat biofortification

Molecular markers serve as a powerful breeding tool to accelerate the development of new crop varieties, particularly for nutrient enrichment (Grewal et al., 1996). While several QTL related to Zn have been identified, few molecular markers have been developed for these traits, and even fewer are suitable for practical breeding applications. For example, Sadeghzadeh (2010) developed a molecular marker based on *sznr1* (seed Zn regulator‐1) on the 2H chromosome of barley, enabling marker‐assisted selection (MAS) to improve barley's productivity and nutritional quality under zinc‐deficient conditions. Despite the identification of wheat orthologs of Zn homeostasis genes from model plants, a lack of functional markers has hindered biofortification breeding (Tong et al., 2020, 2022). In this study, the KASP marker *KASP‐AX‐108829087* was validated across 100 local wheat cultivars, confirming its potential usefulness in MAS for wheat biofortification. This marker ensures robustness and reliability in selecting for GrZnc enhancement.

New breeding strategies aim to address future agricultural challenges, such as selecting elite materials from wild or semi‐wild plant species (Zhang et al., 2021), followed by the rapid introduction of domestication‐related traits using genetic tools while retaining desired characteristics (Li et al., 2018; Yu & Li, 2022; Zhang et al., 2023; Zsögön et al., 2018). The *Gpc‐B1* gene, encoding an NAC transcription factor from ancestral wild wheat, influences grain protein, Zn, and iron content and has been successfully incorporated into breeding programs (Uauy et al., 2006). The groundcherry (*Physalis pruinosa*), a distant relative of tomato, can be rapidly improved by editing tomato domestication genes (Lemmon et al., 2018). Similarly, *QGrZnc.sau‐AM‐4A*, a major and stably expressed QTL for GrZnc, was identified from a wild species and validated across different genetic backgrounds. Furthermore, *QGrZnc.sau‐AM‐4A* does not appear to cause dilution effects. The candidate genes within this interval can be further explored through fine mapping and map‐based cloning, and introduced into modern wheat cultivars via gene editing technology to achieve “pre‐Green Revolution” levels of Zn in grains (Velu et al., 2019). The insights gained from genes controlling Zn in wheat can be applied to other crops through gene homology (Ghandilyan et al., 2006). Collectively, these findings suggest that *QGrZnc.sau‐AM‐4A* represents a valuable resource for Zn biofortification in wheat.

## 5 CONCLUSIONS

A major and stably expressed QTL, *QGrZnc.sau‐AM‐4A*, controlling GrZnc, was identified in tetraploid wheat and validated across different genetic backgrounds. We analyzed the correlations between GrZnc and other agronomic traits, predicting a candidate gene involved in metal transport within the QTL interval. These findings, along with the KASP marker linked to the QTL, will aid in understanding the genetic basis of GrZnc and its incorporation into breeding programs.

## AUTHOR CONTRIBUTIONS

**Zhaoyong Zeng**: Investigation; methodology; software; validation; writing—original draft. **Jian Ma**: Methodology; resources; software; supervision; validation; writing—review and editing. **Bingjie Chen**: Data curation; formal analysis; methodology. **Huaping Tang**: Formal analysis; methodology; resources; software; visualization. **Xin Xian**: Software; validation. **Yuanfeng Huo**: Resources; software; writing—review and editing. **Yinggang Xu**: Formal analysis; resources; software. **Xiaoyan Tang**: Resources; software; supervision; validation. **Xuesong Gao**: Formal analysis; methodology; software; visualization. **Guangdeng Chen**: Conceptualization; data curation; formal analysis; funding acquisition; methodology; resources; supervision; visualization; writing—review and editing.

## CONFICT OF INTEREST STATEMENT

All authors declare no conflicts of interest.

## Supporting information




**Table S1** Basic physiochemical properties of soil before planting.
**Table S2** Details of the primer sequences of the specific KASP marker.
**Table S3** Ecological location for measuring spike length (SL), spike density (SD), spikelet number per spike (SNS), kernel number per spikelet (KNS), thousand kernel weight (TKW), and grain yield (GY) in AM population.
**Table S4** Analysis of variance for grain zinc concentration at five environments in AM population.

**Fig. S1** Correlation between GrZnc and other agronomic traits in the AM population. GrZnc: grain zinc concentration; SL: spike length, SD: spike density; SNS: spikelet number per spike; KNS: kernel number per spikelet; TKW: thousand kernel weight; GY: grain yield.

**Fig. S2** Physical interval of *QGrZnc.sau‐AM‐4A* and the predicted genes. Dotted line indicates the corresponding orthologs.

**Fig. S3** Analysis of spatiotemporal expression patterns of some genes within the interval.

**Fig. S4** Logarithm of odds (LOD) value of conditional QTL for grain zinc concentration (GrZnc). (a). Student's t‐test for the two groups of lines carrying the allele from either AL or LM001 at *QGrZnc.sau‐AM‐4A* from the AM recombinant inbred line (RIL) population for plant height (PH) (b).

**Fig. S5** Conditional quantitative trait loci analysis of grain zinc concentration (GrZnc) (a). Student's t‐test for the two groups of lines carrying the allele from either AL or LM001 at *QGrZnc.sau‐AM‐4A* from the AM recombinant inbred line (RIL) population for thousand kernel weight (TKW) (b).

## Data Availability

All data generated or analyzed during this study are included in this published article and its supplementary information files, further inquiries can be directed to the corresponding author.
