# Supplementary material for: A novel, major, and validated QTL for grain zinc concentration independent of yield traits in tetraploid wheat
Source: Plant Genome. 2025 Apr 23;18(2):e70029. doi: 10.1002/tpg2.70029 (PMC12018297; doi:10.1002/tpg2.70029)
Supplement: Supplementary file 1 [file TPG2-18-e70029-s002.docx]

**Table S1** Basic physiochemical properties of soil before planting.

| **Indexes** | **pH** | **Total nitrogen (g·kg^-1^)** | **Alkali-hydrolyzable nitrogen (mg·kg^-1^)** | **Available phosphorus (mg·kg^-1^)** | **Available potassium (mg·kg^-1^)** | **Organic matter (g·kg^-1^)** | **Available zinc (mg·kg^-1^)** |
| --- | --- | --- | --- | --- | --- | --- | --- |
| WeJiang | 6.8 | 1.25 | 121.21 | 18.52 | 133.36 | 28.36 | 2.16 |
| ChongZhou | 7.11 | 1.02 | 106.01 | 15.09 | 113.04 | 23.12 | 2.11 |
| YaAn | 6.95 | 1.44 | 130.01 | 13.3 | 152.01 | 26.15 | 2.04 |

**Table S2** Ecological location for measuring spike length (SL), thousand-kernel weight (TKW), kernel number per spikelet (KNS), spikelet number per spike (SNS), spike density (SD), and grain yield (GY) in AM population.

| **Population** | **Traits** | **Environments** | | | | | | | |
| --- | --- | --- | --- | --- | --- | --- | --- | --- | --- |
| AM | SL | 2017CZ^1^ | 2018CZ^1^ | 2019CZ^1^ | 2020CZ^1^ | 2020WJ^1^ | 2020YA^1^ | 2021CZ^1^ | 2021WJ^1^ |
|  | PH | 2017CZ^1^ | 2018CZ^1^ | 2019CZ^1^ | 2020CZ^1^ | 2020WJ^1^ | 2020YA^1^ | 2021CZ^1^ | 2021WJ^1^ |
|  | TKW | 2019CZ^1^ | 2020CZ^1^ | 2020WJ^1^ | 2020YA^1^ | 2021CZ^1^ | 2021WJ^1^ |  |  |
|  | KNS | 2019CZ^1^ | 2020CZ^1^ | 2020WJ^1^ | 2020YA^1^ | 2021CZ^1^ | 2021WJ^1^ |  |  |
|  | SNS | 2017CZ^1^ | 2018CZ^1^ | 2019CZ^1^ | 2020CZ^1^ | 2020WJ^1^ | 2020YA^1^ | 2021CZ^1^ | 2021WJ^1^ |
|  | SD | 2017WJ^2^ | 2017CZ^2^ | 2017YA^2^ | 2018WJ^2^ | 2018CZ^2^ | 2018YA^2^ | 2018KB^2^ |  |
|  | GY | 2020WJ^1^ | 2021CZ^1^ | 2021WJ^1^ |  |  |  |  |  |

Note:
Population: AM: Ailanmai/LM001

[1] Mo Z., Zhu J., Wei J., Zhou J., Xu Q., Tang H., Mu Y., Deng M., Jiang Q., Liu Y., Chen G., Wang J., Qi P., Li W., Wei Y., Zheng Y., Lan X., &Ma J (2021) The 55K SNP-based exploration of QTLs for spikelet number per spike in a tetraploid wheat (*Triticum turgidum* L.) Population: Chinese Landrace "Ailanmai" × Wild Emmer. *Frontiers in Plant Science*, 12:732837. https://doi.org/10.3389/fpls.2021.732837.

[2] You, J., H. Liu., S. Wang., W. Luo., L. Gou., H. Tang., Y. Mu., M. Deng., Q. Jiang., G. Chen., P. Qi., Y. Peng., L. Tang., A. Habib., Y. Wei., Y. Zheng., X. Lan & J. Ma. (2021). Spike density quantitative trait loci detection and analysis in tetraploid and hexaploid wheat recombinant inbred line populations. *Frontiers in Plant Science*, 12, 796397. https://doi.org/10.3389/fpls.2021.796397.

**Table S3** Details of the primer sequences of the specific KASP markers

| **Primer name** | **Tag** | **Primer sequence (5'-3')** | **IWGSC RefSeq v2.1Position** |
| --- | --- | --- | --- |
| KASP-AX-108829087 | F-FAM | GAAGGTGACCAAGTTCATGCTCATCAATAGAAAAAACATGGCATC | 60.02 Mbp |
|  | F-HEX | GAAGGTCGGAGTCAACGGATTCATCAATAGAAAAAACATGGCATT |  |
|  | R | TGTTGTTAGTGTCTTCTCAGCCAGG |  |

**Table S4** Analysis of variance for grain zinc concentration at five environments in AM population.

| **Traits** | **Source** | **DF** | **MS** | **F-value** | ***P*-value** |
| --- | --- | --- | --- | --- | --- |
| Grain zinc concentration | Block/Environment | 5 | 2.4979 | 20.6061 | <0.001 |
|  | Genotype | 120 | 1368.58 | 11290 | <0.001 |
|  | Environment | 4 | 6103.95 | 50354.2 | <0.001 |
|  | GE-interaction | 414 | 513.645 | 4237.29 | <0.001 |

DF: Degrees of freedom; MS: Mean square.
