# Supplementary figures and images for: A novel, major, and validated QTL for grain zinc concentration independent of yield traits in tetraploid wheat
Source: Plant Genome. 2025 Apr 23;18(2):e70029. doi: 10.1002/tpg2.70029 (PMC12018297; doi:10.1002/tpg2.70029)

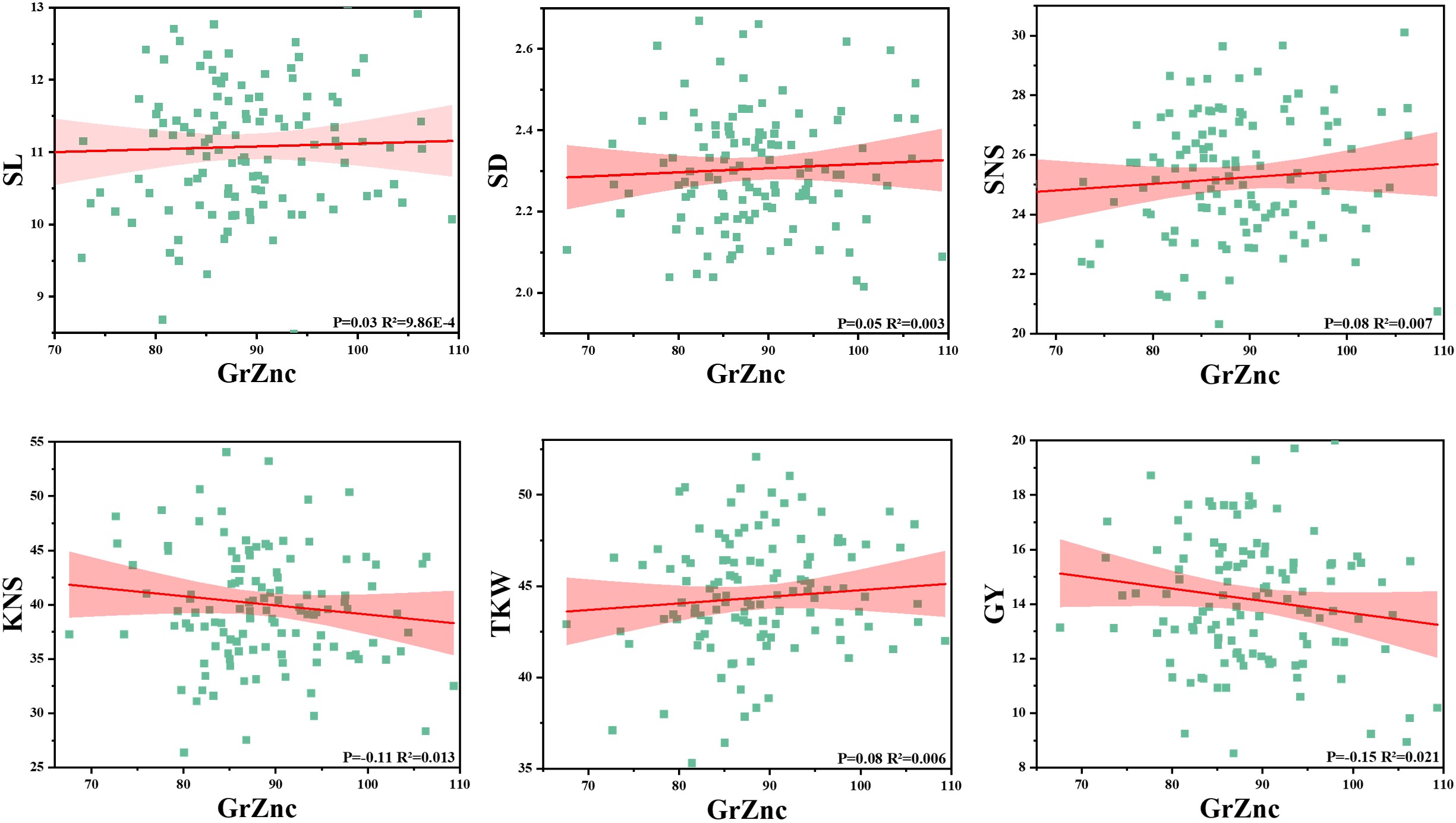

Supplement: Supplementary file 2 — Fig. S1 Correlation between GrZnc and other agronomic traits in the AM population. GrZnc: grain zinc concentration; SL: spike length, SD: spike density; SNS: spikelet number per spike; KNS: kernel number per spikelet; TKW: thousand kernel weight; GY: grain yield. [file TPG2-18-e70029-s001.jpg]

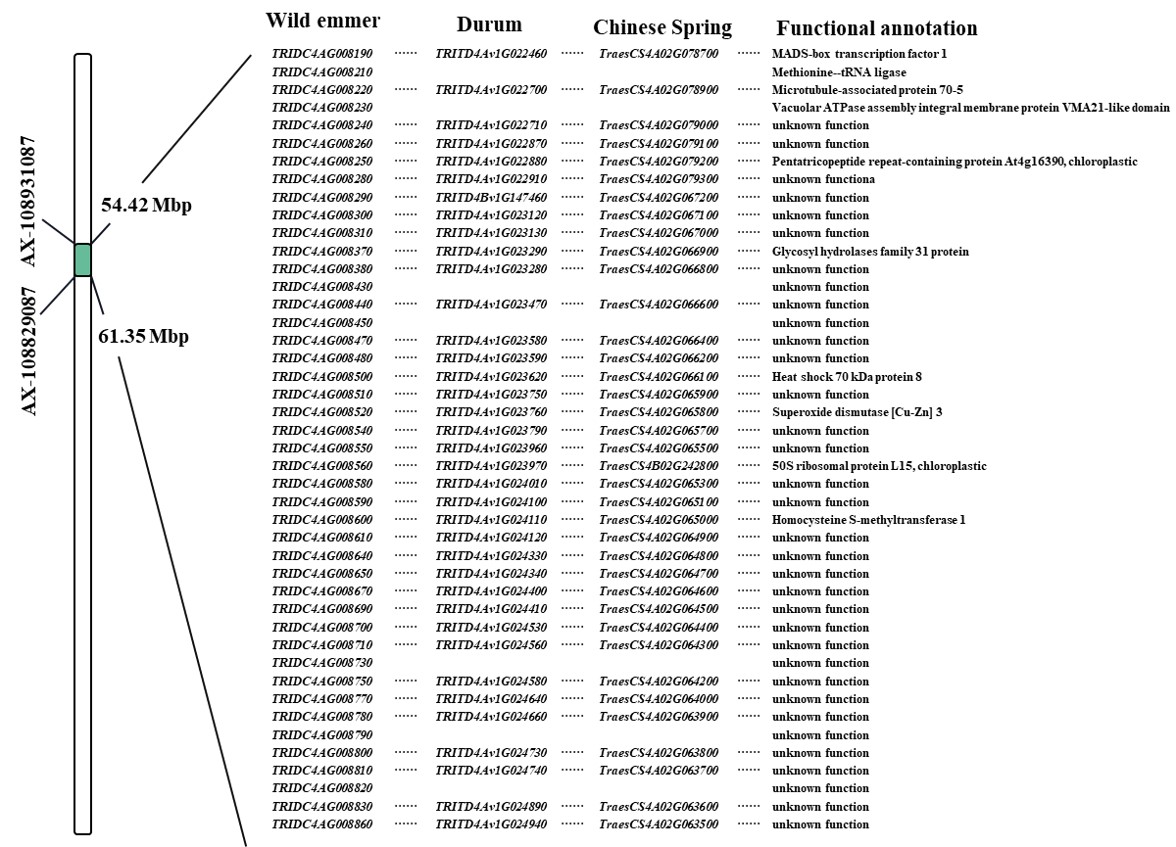

Supplement: Supplementary file 3 — Fig. S2 Physical interval of QGrZnc.sau‐AM‐4A and the predicted genes. Dotted line indicates the corresponding orthologs. [file TPG2-18-e70029-s006.jpg]

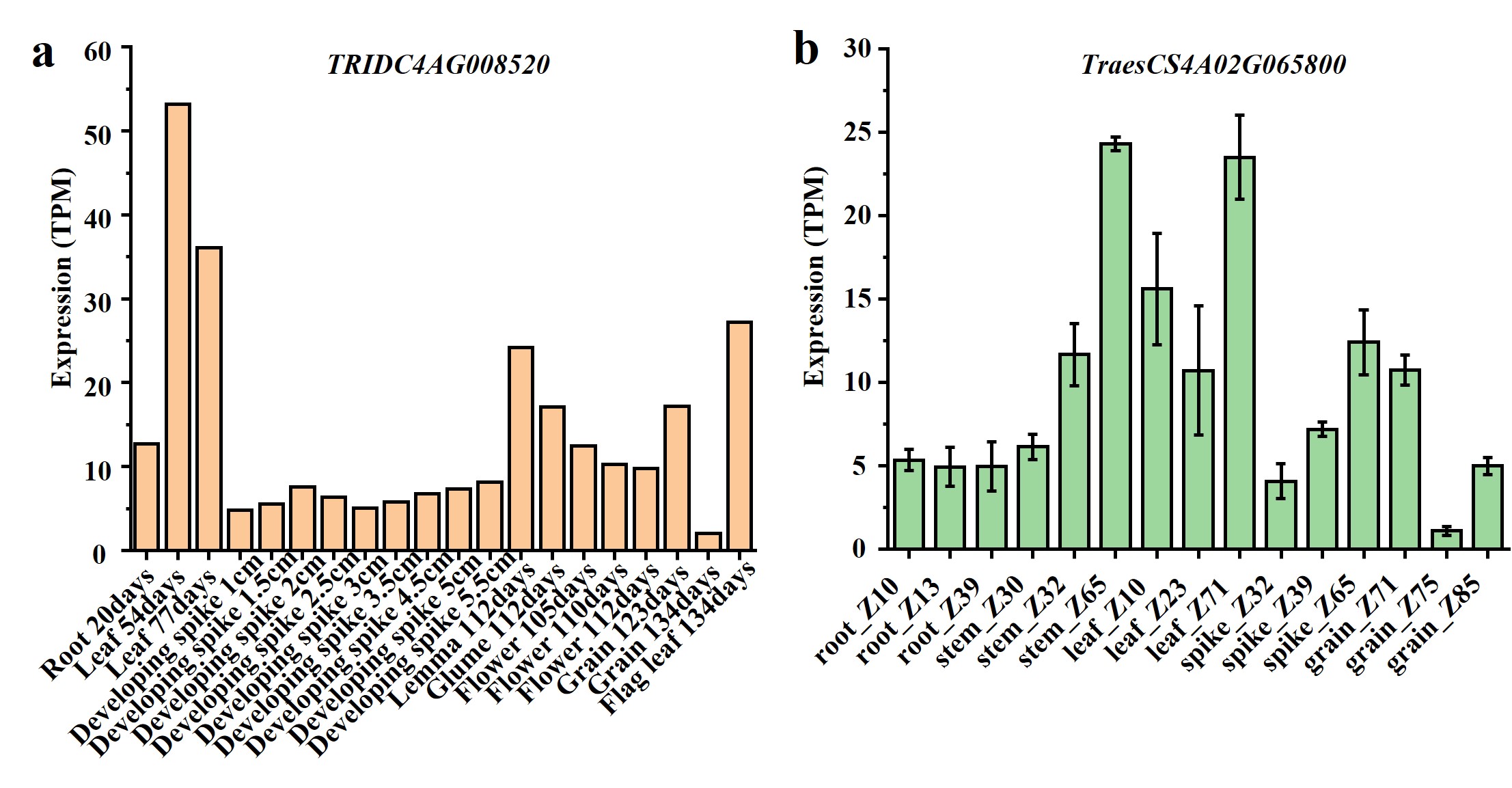

Supplement: Supplementary file 4 — Fig. S3 Analysis of spatiotemporal expression patterns of some genes within the interval. [file TPG2-18-e70029-s003.jpg]

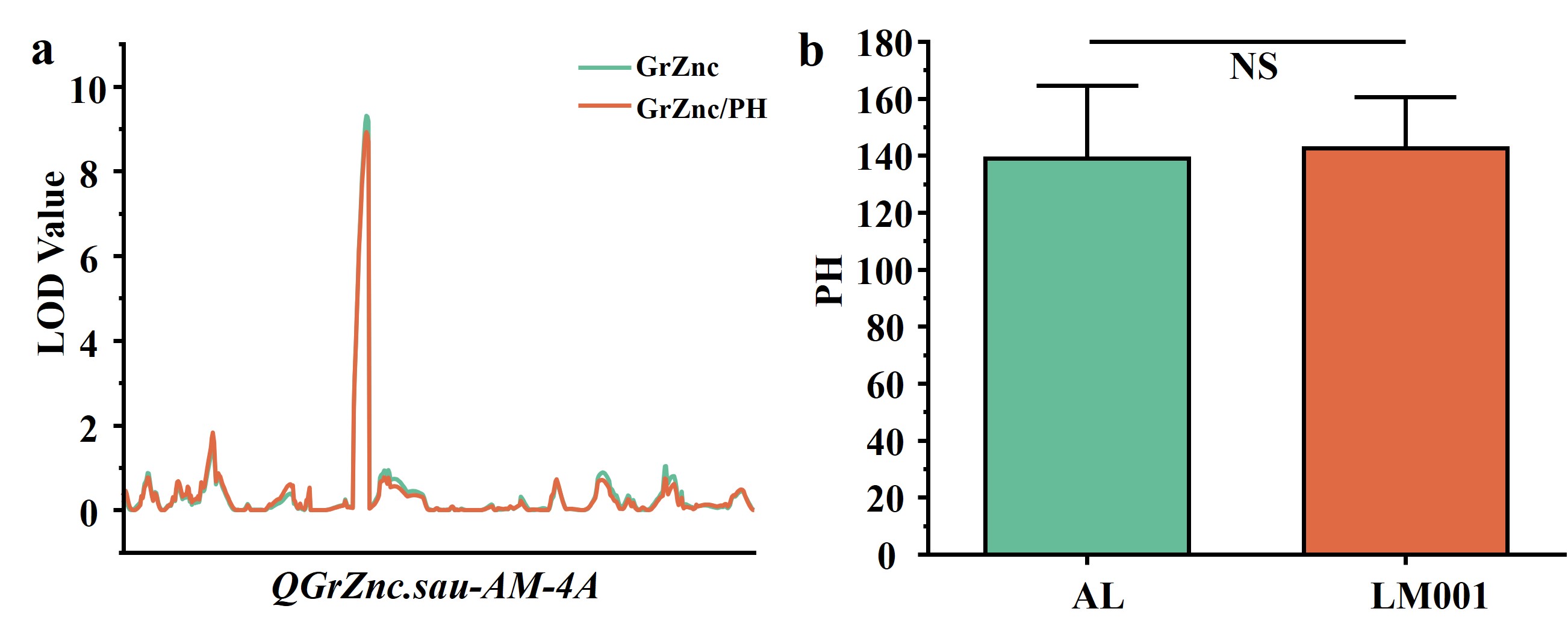

Supplement: Supplementary file 5 — Fig. S4 Logarithm of odds (LOD) value of conditional QTL for grain zinc concentration (GrZnc). (a). Student's t‐test for the two groups of lines carrying the allele from either AL or LM001 at QGrZnc.sau‐AM‐4A from the AM recombinant inbred line (RIL) population for plant height (PH) (b). [file TPG2-18-e70029-s004.jpg]

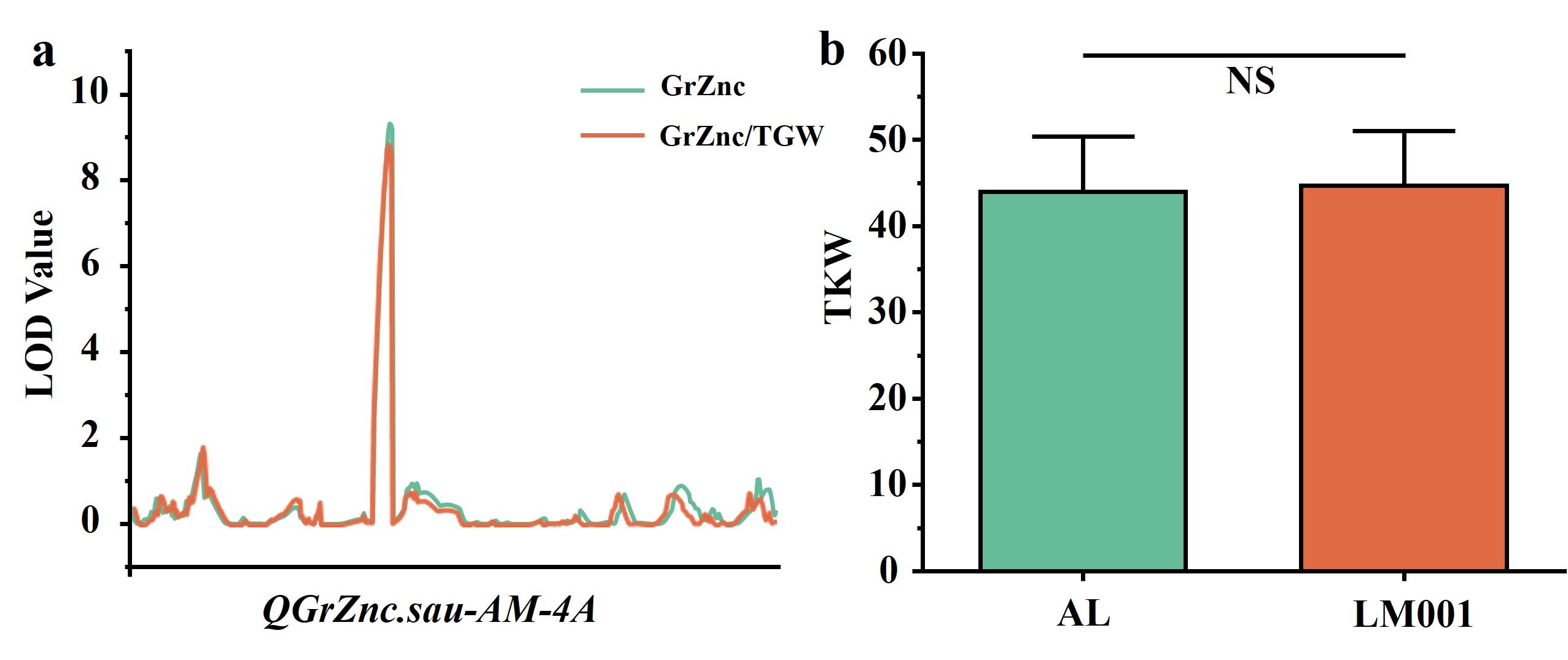

Supplement: Supplementary file 6 — Fig. S5 Conditional quantitative trait loci analysis of grain zinc concentration (GrZnc) (a). Student's t‐test for the two groups of lines carrying the allele from either AL or LM001 at QGrZnc.sau‐AM‐4A from the AM recombinant inbred line (RIL) population for thousand kernel weight (TKW) (b). [file TPG2-18-e70029-s005.jpg]
